# The positive correlation between amphiregulin and insulin resistance

**DOI:** 10.1530/EC-24-0580

**Published:** 2025-03-04

**Authors:** Yun Zhao, Ting Lu, Mian Wu, Xingna Hu, Rong Xiang, Min Feng, Honghong Lu

**Affiliations:** ^1^ Department of Endocrinology, The Affiliated Suzhou Hospital of Nanjing Medical University, Suzhou Municipal Hospital, Gusu School, Nanjing Medical University, Suzhou, Jiangsu, P.R. China

**Keywords:** amphiregulin, insulin resistance, type 2 diabetes mellitus, obesity

## Abstract

**Objective:**

Obesity and insulin resistance carry a high risk of progressing to type 2 diabetes mellitus. A lot of evidence has tightly associated insulin resistance with chronic inflammation. Besides, it has been reported that the activation of amphiregulin epidermal growth factor receptor pathways is involved in chronic inflammation. The aim of this study was to evaluate the relationship between insulin resistance and amphiregulin.

**Methods:**

Data from 203 volunteers were collected from November 2020 to June 2023 visiting the Affiliated Suzhou Hospital of Nanjing Medical University, Jiangsu, China. The serum levels of amphiregulin and diabetes-related parameters were measured in all participants. The correlation analysis and multiple stepwise regression of amphiregulin and some diabetes indicators were performed in all groups.

**Results:**

The concentrations of amphiregulin were 143.29, 163.29, 158.92, 171.89 and 155.03 pg/mL in the normal, obesity, nonobese diabetes, obese diabetes and obese diabetes after therapy groups (*P* < 0.01), respectively, and the homeostasis model assessment index were 1.6, 4.01, 3.93, 6.67 and 3.4, respectively (*P* < 0.01). Moreover, amphiregulin positively correlated with the homeostasis model assessment index in each separated group and the total sample (*r* = 0.644, *P* < 0.01). Meanwhile, the regression analysis showed a strong, positive association between amphiregulin and the homeostasis model assessment index (*P* < 0.01). More importantly, this correlation remained after obese diabetes patients were treated with drugs to relieve insulin resistance.

**Conclusion:**

Amphiregulin is upregulated in obese individuals than in normal size people, whether diabetic or not, and positively correlates with the homeostasis model assessment index, suggesting early signs of insulin resistance and abnormal glucose metabolism.

## Introduction

Obesity is a global pandemic and one of the most important public health concerns in many countries (1). It is often accompanied by many metabolic syndromes such as diabetes, hypertension, dyslipidemia and nonalcoholic fatty liver disease (NAFLD). Excessive nutrients subsequently result in the occurrence of insulin resistance, putting people in danger of abnormal glucose and lipid metabolism (2, 3). It is well-known that insulin resistance may occur a few years before the development of type 2 diabetes mellitus (T2DM) (4). At the early stage in T2DM, pancreatic β-cells try their best to increase insulin secretion to maintain normoglycemia. Over time, this compensatory response would gradually weaken or even lose, yielding overt hyperglycemia (5). Nowadays, it is no longer enough to diagnose T2DM and treat patients with classical drugs, but rather take action to predict the onset of diabetes, give guidance to those with prediabetes and prevent the progression of disease and its complications.

Many findings have implied that the gradual development of T2DM might be reversed by prevention of insulin resistance (6, 7). Given the increasing incidence of T2DM in obese people, there is an urgent need for alternative tools to identify person at risk of insulin resistance in the community. Our study found that amphiregulin (AREG) was upregulated when insulin resistance occurred at the early state of T2DM. AREG was initially identified as a member of the epidermal growth factor (EGF) family, which participated in various physiological processes such as cell proliferation, differentiation and regeneration by its binding to the epidermal growth factor receptor (EGFR) (8, 9). Emerging data suggest that the activation of downstream AREG-EGFR pathways including mitogen-activated protein kinases (MAPKs), phosphatidylinositide 3-kinases (PI3Ks)/protein kinase B (AKT) and mammalian target of rapamycin (mTOR) is involved in chronic infection and inflammation (10, 11, 12). Moreover, many pieces of evidence have tightly associated insulin resistance with chronic inflammatory responses when glucose tolerance is impaired by the activation of multiple inflammatory signaling pathways (13, 14, 15).

Recently, Raugh *et al.* reported that the AREG/EGFR axis had limited contribution in controlling autoimmune diabetes (16). But the involvement of AREG in insulin resistance or T2DM has not yet been established. We hypothesized that AREG might play a role in insulin resistance at the early stage in T2DM. Thus, the study aimed to measure the serum levels of AREG in obesity and patients with T2DM and compare them with healthy individuals, attempting to reveal the relationship between AREG and insulin resistance.

## Materials and methods

### Study design and population

Inclusion criteria: the sample was made up of volunteers visiting the Affiliated Suzhou Hospital of Nanjing Medical University, Jiangsu, China, from November 2020 to June 2023. All participants were aged ≥18 years. T2DM was diagnosed within three months according to the 1999 diagnostic criteria of the World Health Organization (WHO) and not taking any hypoglycemic drugs. All subjects should participate voluntarily and actively cooperate. Exclusion criteria were the history of T1DM, special types of diabetes, prediabetes, acute infections, history of chronic kidney or cardiovascular diseases and malignancy. The total sample was finally divided into four groups based on body mass index (BMI) and blood glucose: the normal group, the obese group, the nonobese diabetes group and the obese diabetes group (obesity is defined as BMI ≥28 kg/m^2^). Subsequently, the obese diabetes people were treated with metformin or glucagon-like peptide-1 receptor agonist liraglutide or both for three months.

Each volunteer provided his written informed consent. All procedures and secondary analyses were approved by the medical ethics committee of Suzhou Municipal Hospital (protocol number is KL901115, 2020) and conducted according to the Nuremberg Code and the Declaration of Helsinki.

### Biochemical analysis

The level of serum fasting glucose (FBG) was estimated by an Accu-Chek III glucose analyzer. Fasting serum insulin (FPI) was measured by an ELISA kit (Santa Cruz Biotechnology Inc. Shanghai Universal Biotech, China). Insulin resistance was assessed by the homeostasis model assessment index (HOMA-IR). HOMA-IR = (FPG (mmol/L) × FPI (IU/mL))/22.5 (6).

### Enzyme-linked immunosorbent assay (ELISA)

All blood samples were collected by venipuncture and clotted for 30 min at room temperature. Then, these samples were separated by centrifugation at 3000 rpm for 15 min and distributed in sterile tubes for storage at −80°C until analysis. They needed to return to room temperature before measurement. The concentration of AREG in serum was quantified by the specific human AREG ELISA kits (catalog number: 106029, Enzyme-linked Biotechnology Co., Ltd, China), according to the manufacturer’s protocol. The detection range was 31.25–1000 pg/mL and the normal range was 67.5–176.45 pg/mL. Each sample was tested in duplicate.

### Statistical analysis

Data were analyzed using the SPSS v.22.0 statistical software and first tested for normal distribution. Normally distributed data were expressed as the mean ± standard deviation and the non-normally distributed data were expressed as the median (interquartile spacing). Comparisons between groups were made by one-way ANOVA test. The association between paired variables was analyzed by Pearson and Spearman correlation analysis. Multiple stepwise regression analyses were used to assess the relationship between AREG and diabetes-related confounders. *P* values <0.05 were considered statistically significant.

## Results

### Clinical characteristics

**Figure 1:**
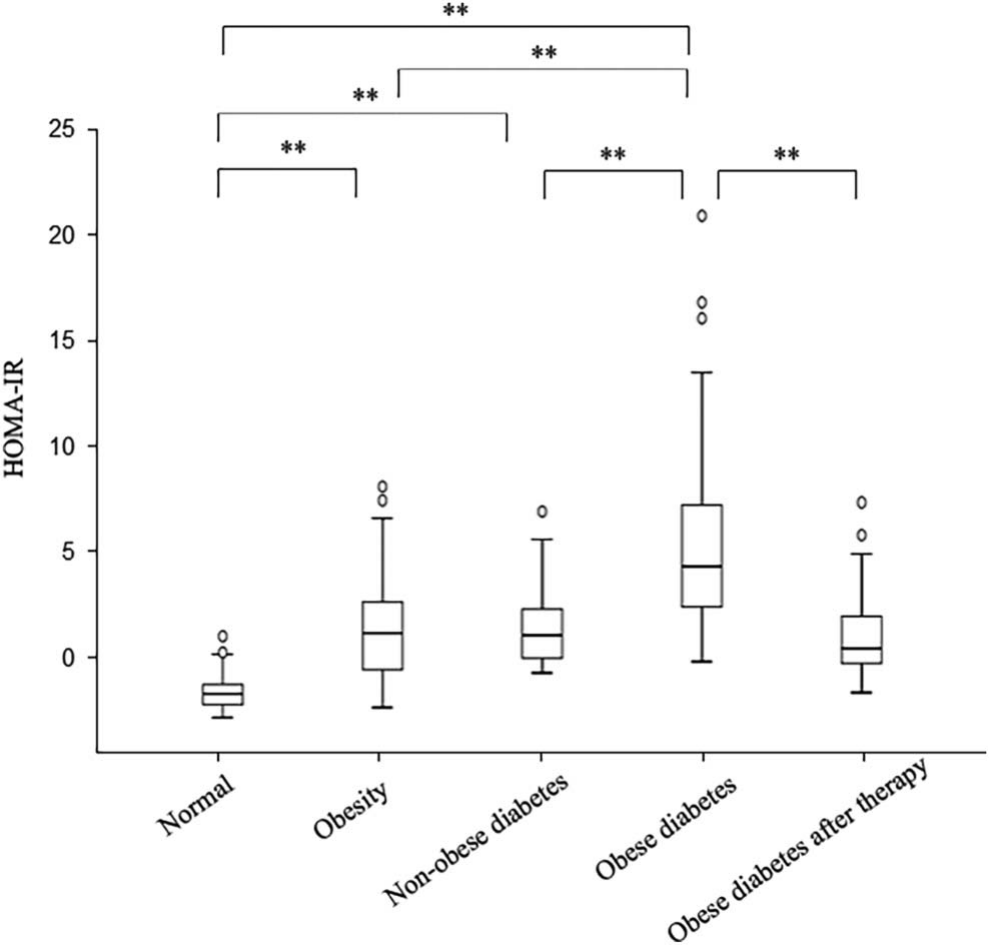
Comparison of HOMA-IR levels in different groups. **P < 0.01; *P < 0.05.

**Figure 2:**
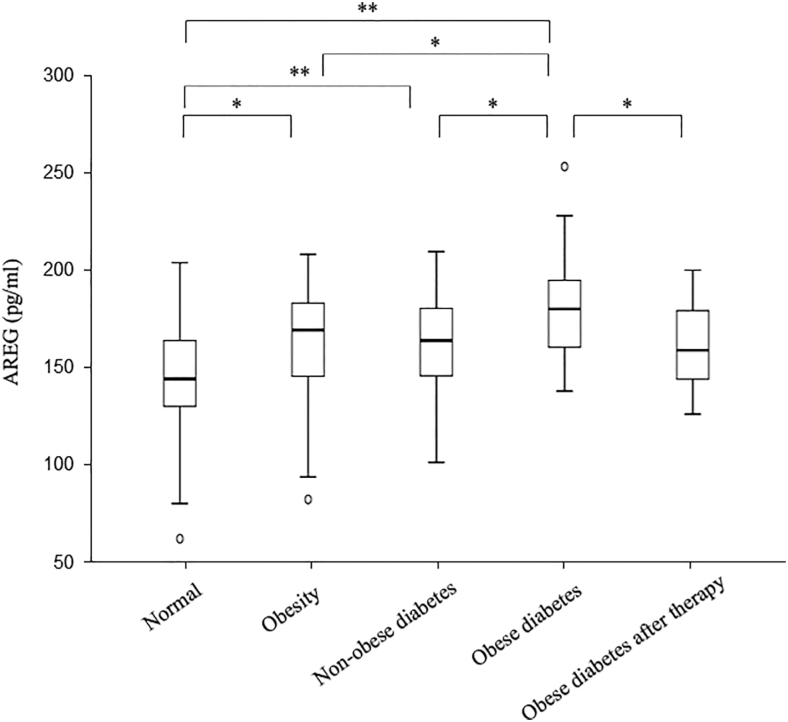
Comparison of AREG serum levels in different groups. **P < 0.01; *P < 0.05.

The comparison of demographic and biochemical indicators among control, the obesity, the nonobese diabetes, the obese diabetes and the obese diabetes after therapy groups is presented in Table 1. There were no significant differences in sex, age and creatinine among five groups. When compared with the normal group, the obesity and the obese diabetes groups had significantly higher levels of BMI, FPI, HOMA-IR, alanine aminotransferase (ALT), AREG, serum uric acid (SUA) and triglyceride (TG) (all *P* < 0.05, Table 1, Figs 1 and 2). Besides, the obesity group showed lower levels of diastolic blood pressure (DBP), FBG, HOMA-IR, AREG, ALT, aspartate aminotransferase (AST), creatinine (Cr), SUA, total cholesterol (TC) and TG compared to the obese diabetes group (all *P* < 0.05, Table 1). Meanwhile, the obese diabetes group also had higher results of BMI, DBP, FPI, HOMA-IR, AREG, ALT, AST, SUA, TC and TG than the nonobese diabetes group (all *P* < 0.05, Table 1). In addition, the subjects of nonobese diabetes group had higher FBG, FPI, HOMA-IR and AREG than did the control group subjects (all *P* < 0.05, Table 1). More importantly, when the obese diabetes patients were treated with drugs (metformin or liraglutide or both), the patients after therapy had lower targets of BMI, systolic blood pressure (SBP), DBP, FBG, FPI, HOMA-IR, AREG, SUA and TC (all *P* < 0.05, Table 1).

**Table 1:** Comparison of clinical characteristics of study participants in different groups.

Variables	Normal (53)	Obesity (51)	Nonobese diabetes (48)	Obese diabetes (51)	Obese diabetes after therapy (51)	*P*
Male/female	25/28	23/28	26/22	28/23	28/23	0.771
Age (years)	42.72 ± 14.29	38.08 ± 11.91	43.58 ± 11.39	39.80 ± 11.81	39.80 ± 11.81	0.145
BMI (kg/m2)	22.04 ± 1.40	33.37 ± 4.38*	22.56 ± 1.23^†^^,^^§^	33.40 ± 4.05*^,^^‡^	30.75 ± 3.10*^,^^†^^,^^‡^^,^^§^	<0.001
SBP (mmHg)	130.42 ± 13.75	135.27 ± 12.99	137.33 ± 13.44	140.35 ± 14.71*	132.49 ± 10.92^§^	0.002
DBP (mmHg)	76.58 ± 7.57	78.59 ± 8.75^§^	77.40 ± 10.49^§^	83.20 ± 10.34*^,^^†^^,^^‡^	76.51 ± 6.68^§^	0.001
FBG (mmol/L)	5.29 ± 0.46	5.20 ± 0.64^‡^^,^^§^	9.09 ± 1.69*^,^^†^	10.07 ± 3.08*^,^^†^	7.39 ± 1.52*^,^^†^^,^^‡^^,^^§^	<0.001
FPI (IU/mL)	6.77 (5.07, 8.27)	17.28 (11.40, 26.31)*^,^^‡^	9.26 (8.18, 12.31)*^,^^†^^,^^§^	16.79 (10.38, 22.03)*^,^^‡^	10.45 (9.05, 14.28)*^,^^†^^,^^§^	<0.001
HOMA-IR	1.60 (1.17, 2.03)	4.01 (2.54, 5.26)*^,^^§^	3.93 (3.01, 5.00)*^,^^§^	6.67 (4.94, 9.14)*^,^^†^^,^^‡^	3.40 (2.77, 4.72)*^,^^§^	<0.001
AREG (ng/mL)	143.29 (128.83, 159.12)	163.29 (144.26, 174.88)*^,^^§^	158.92 (144.19, 172.18)*^,^^§^	171.89 (156.28, 184.07)*^,^^†^^,^^‡^	155.03 (143.09, 172.19)*^,^^§^	<0.001
ALT (IU/L)	21.5 (16.85, 36.85)	36.1 (22.9, 45.3)*^,^^‡^^,^^§^	20.85 (15.10, 37.85)^†^^,^^§^	52.1 (41.7, 106.7)*^,^^†^^,^^‡^	43.3 (32.1, 66.2)*^,^^‡^	<0.001
AST (IU/L)	23.7 (19.15, 30.45)	28.7 (19.4, 39.4)^§^	20.9 (16.8, 27.4)^§^	40.3 (29.7, 69.3)*^,^^†^^,^^‡^	36.0 (25.7, 51.0)*^,^^‡^	<0.001
Cr (μmol/L)	59.48 ± 16.79	59.66 ± 15.52^§^	64.67 ± 17.53	67.34 ± 15.71	63.77 ± 13.85	0.058
SUA (μmol/L)	352.38 ± 85.70	415.89 ± 101.29*^,^^‡^^,^^§^	345.06 ± 100.39^†^^,^^§^	467.48 ± 98.69*^,^^†^^,^^‡^	407.50 ± 63.84*^,^^‡^^,^^§^	<0.001
TC (mmol/L)	4.42 ± 1.12	4.66 ± 1.15^§^	4.67 ± 1.59^§^	5.32 ± 1.22*^,^^†^^,^^‡^	4.55 ± 0.90^§^	0.003
TG (mmol/L)	1.34 (0.98, 2.35)	1.84 (1.28, 2.55)*^,^^‡^^,^^§^	1.38 (1.10, 1.88)^†^^,^^§^	2.37 (1.87, 4.21)*^†^^,^^‡^	2.03 (1.36, 2.54)*^,^^‡^	<0.001

Abbreviations: BMI, body mass index; SBP, systolic blood pressure; DBP, diastolic blood pressure; FBG, fasting blood glucose; FPI, fasting serum insulin; HOMA-IR, the homeostasis model assessment for insulin resistance; AREG, amphiregulin; AST, aspartate aminotransferase; ALT, alanine aminotransferase; Cr, creatinine; SUA, serum uric acid; TC, total cholesterol; TG, triglyceride. *P* for comparisons among the five groups; *P* values were derived from one-way ANOVA for normally distributed continuous variables, Kruskal–Wallis H test for non-normally distributed continuous variables and from chi-square for categorical variables.

* *P* < 0.05, compared with normal group.

^†^ *P* < 0.05, compared with the obesity group.

^‡^ *P* < 0.05, compared with the nonobese diabetes group.

^§^ *P* < 0.05, compared with the obese diabetes group.

### Univariate and multivariate analysis

**Figure 3:**
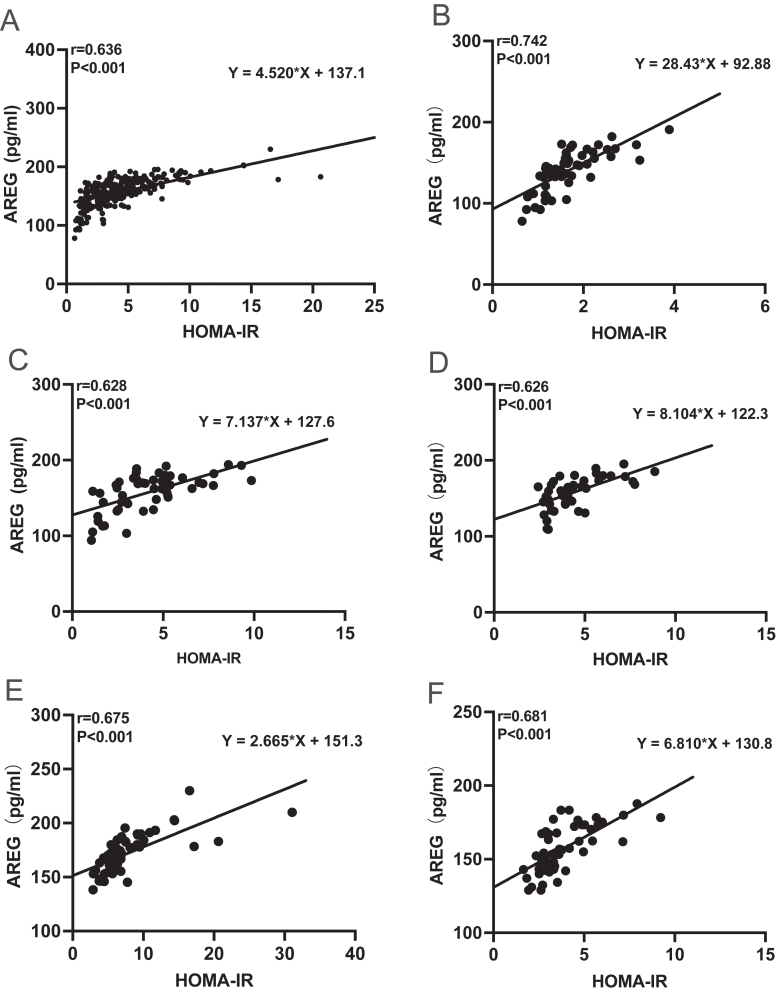
AREG positively correlated with HOMA-IR in total and each group. Linear regression analysis showed the association between AREG and HOMA-IR in human. (A) The total sample; (B) the normal group; (C) the obese group; (D) the nonobese diabetes group; (E) the obese diabetes group; (F) the obese diabetes after therapy group.

Linear regression analysis concerning HOMA-IR or AREG was performed, with HOMA-IR or AREG serving as the dependent variable and other parameters acting as independent variables (Table 2). Pearson correlation analysis showed that AREG was significantly positively correlated with HOMA-IR in each group and the total study population (all *P* < 0.05, Fig. 3).

**Table 2:** Correlation analysis of HOMA-IR and AREG with other parameters in all study population.

	HOMA-IR		AREG	
	*r*	*P* value	*r*	*P* value
Age (years)	0.008	0.901	0.040	0.530
BMI (kg/m^2^)	0.384^†^	0	0.320^†^	0
SBP (mmHg)	0.181^†^	0.004	0.205^†^	0.001
DBP (mmHg)	0.247^†^	0	0.165^†^	0.009
FBG (mmol/L)	0.433^†^	0	0.367^†^	0
FPI (IU/mL)	0.837^†^	0	0.583^†^	0
HOMA-IR	/	/	0.636^†^	0
AREG (pg/mL)	0.636^†^	0	/	/
ALT (IU/L)	0.393^†^	0	0.265^†^	0
AST (IU/L)	0.362^†^	0	0.234^†^	0
Cr (μmol/L)	0.068	0.282	0.065	0.303
SUA (μmol/L)	0.277^†^	0	0.168^†^	0.007
TC (mmol/L)	0.238^†^	0	0.117	0.064
TG (mmol/L)	0.222^†^	0	0.119	0.058

Spearman correlation analysis was used to analyze the correlation between HOMA-IR or AREG and other parameters. Abbreviations are same as in Table 1.

^†^ *P* < 0.01.

### Effect analysis of structural equation modeling variable in each group

The model outcomes showed that only HOMA-IR manifested a positive association with AREG in normal, obesity, obese diabetes and obese diabetes after therapy groups. Besides, HOMA-IR and BMI exhibited a positive connection with AREG in nonobese diabetes group. To sum up, multiple stepwise linear regression analysis showed that the AREG concentrations and HOMA-IR were correlated in each group (Table 3), and this correlation remained after adjustment for age, sex, BMI, SBP, DBP, FBG, FPI, ALT, AST, Cr, SUA, TC and TG (Table 4). These observations supported our hypothesis that AREG positively correlated with insulin resistance whether diabetic or not.

**Table 3:** Multiple regression of various biomarkers vs AREG in different groups.

Group	Variable	*β*	SE	Standard β	*t*	*P*
Normal	HOMA-IR	28.434	3.599	0.742	7.901	<0.001
Obesity	HOMA-IR	7.137	1.265	0.628	5.644	<0.001
Nonobese diabetes	HOMA-IR	8.199	1.806	0.632	4.541	<0.001
	BMI	3.688	1.801	0.229	2.048	0.046
Obese diabetes	HOMA-IR	2.665	0.377	0.711	7.076	<0.001
Obese diabetes after therapy	HOMA-IR	6.810	1.046	0.681	6.508	<0.001

Abbreviations are same as in Table 1.

**Table 4:** Multiple regression of various biomarkers vs AREG after adjustment in all study population.

Variables	Model 1		Model 2	
	*β*	*P*	*β*	*P*
BMI (kg/m^2^)	0.089	0.092	0.116	0.030*
SBP (mmHg)	0.092	0.061	0.088	0.072
FBG (mmol/L)	0.113	0.035*	0.113	0.035*
FPI (IU/mL)	0.166	0.061	0.736	<0.001**
HOMA-IR	0.636	<0.001**	0.625	<0.001**
TC (mmol/L)	−0.037	0.463	−0.043	0.388

Model 1 adjusted for age, sex, BMI, SBP, DBP, FBG, FPI, ALT, AST, Cr, SUA, TC and TG; Model 2 adjusted for age, sex, BMI, SBP, DBP, FPI, ALT, AST, Cr, SUA, TC and TG. Abbreviations are same as in Table 1.

** *P* < 0.01;

* *P* < 0.05.

## Discussion

Nowadays, many surveys have revealed that a growing number of people are overweight or obese (17, 18). These people are at a high risk of developing insulin resistance, glucose intolerance and dyslipidemia. Thus, it is very important and meaningful to identify these sub-healthy persons and change their inappropriate life styles. In this study, we found that the obesity and obese diabetes groups had markedly higher levels of BMI, FPI, HOMA-IR, ALT, SUA, TG and AREG than healthy subjects. Furthermore, circulating levels of AREG were significantly higher in obese diabetes than the obesity, probably owing to the higher demand for insulin in T2DM. Another worthy observation was the higher concentration of AREG in obese individuals vs control individuals, which might turn to be an early alteration in persons with insulin resistance in the long-term development of T2DM. When the obese people have not yet developed to diabetes, AREG has begun to rise when they suffer from insulin resistance. These findings seemed to be in accordance with previous studies, which showed that AREG mRNA and protein expression were markedly increased in response to high-fat diets (9, 19). Somewhat surprisingly, it has also been reported that global AREG deletion protected against insulin resistance, as indicated by lower body weight and FBG, and improved glucose tolerance and insulin tolerance tests (9).

Therefore, necessary interventions should come into operation to avoid obesity proceeding or even diabetes emerging. Then, we treated the obese diabetes with metformin or liraglutide or both drugs, which had been proven to alleviate insulin resistance (15, 20). After three months treatment, the serum level of AREG was declined nearly 10%, accompanied by decreased BMI, SBP, DBP, FBG, FPI, SUA, TC and HOMA-IR, indicating promising weight loss, improved metabolism disorders and recovered insulin sensitivity. Our study showed that the serum AREG concentration was significantly and positively correlated with HOMA-IR after adjustment for diabetes-related variables. As shown in Fig. 3F, the correlation between HOMA-IR and AREG remained after therapy.

AREG is generally recognized as a member of the EGF family (8). A number of studies have suggested its critical role in chronic inflammatory responses (21, 22). However, to our knowledge, very limited research has been done on the possible role of AREG in glucose metabolic disorders. Heo *et al.* demonstrated that amphiregulin participated in hepatic inflammation through nuclear factor kappa-B (NF-κB) and MAPK signaling by activating inducible nitric oxide synthase (iNOS) and cyclooxygenase-2 expression (19). Another study reported that AREG regulated odontogenic differentiation of dental pulp stem cells by the activation of MAPK pathway (11). These inflammatory signaling pathways are also excessively activated at the early stage of diabetes, contributing to insulin resistance (13, 14, 15). With regard to the mechanism involved, we proposed the hypothesis that AREG might play a role in insulin resistance partly through AREG-EGFR pathways. Recently, Savage *et al.* indicated that Treg cell-derived AREG promotes glucose intolerance in a NASH-dependent manner through EGFR signaling and IL-6, which is consistent with our hypothesis (23). Future studies will be required to investigate the detailed mechanisms of AREG in modulating insulin sensitivity.

The strengths of this study included that we revealed the early alteration of AREG when obese individuals suffered from insulin resistance. Moreover, after three months follow-up, the serum levels of AREG decreased, accompanied by the alleviation of insulin resistance. Undeniably, our study had some limitations: First, the sample size was relatively small, and our study subjects were restricted to volunteers in Suzhou, Jiangsu, China. Our future studies will attempt to address this limitation by expanding sample size and obtaining adequate representation of people in different regions. Second, AREG is influenced by many factors, such as infection, inflammation, tissue damage and cancer (23). Therefore, when we discuss the relationship between AREG and insulin resistance, we should exclude the abovementioned situations. Third, more detailed biological mechanisms between AREG and insulin resistance are needed to find out.

In conclusion, AREG was upregulated in obese individuals than in normal size people, whether diabetic or not, and was significantly and positively correlated with HOMA-IR, suggesting early signs of insulin resistance and abnormal glucose metabolism.

## Declaration of interest

The authors declare that there is no conflict of interest that could be perceived as prejudicing the impartiality of this work.

## Funding

This work did not receive any specific grant from any funding agency in the public, commercial or not-for-profit sector.

## Author contribution statement

Yun Zhao and Honghong Lu designed the study. Mian Wu, Xingna Hu and Rong Xiang interpreted the data and conducted the analysis. Yun Zhao and Ting Lu drafted and edited the manuscript. Min Feng approved the manuscript and was the guarantor of this work and took responsibility for the integrity of the data. All authors read and approved the final manuscript.
